# Deformation Twinning in Polycrystalline Mg Microstructures at High Strain Rates at the Atomic Scales

**DOI:** 10.1038/s41598-019-39958-w

**Published:** 2019-03-05

**Authors:** Garvit Agarwal, Avinash M. Dongare

**Affiliations:** Department of Materials Science and Engineering and Institute of Materials Science, University of Connecticut, Storrs, CT 06269 USA

## Abstract

Large scale molecular dynamics (MD) simulations are carried out to investigate the twinning behavior as well as the atomic scale micromechanisms of growth of tension and compression twins in polycrystalline Mg microstructures at high strain rates. A new defect characterization algorithm (extended-common neighbor analysis (E-CNA)) is developed that allows for an efficient identification of various types of twins in HCP microstructures. Unlike other local orientation analysis methods, the E-CNA method allows for atomic scale characterization of the structure of different types of twin boundaries in HCP microstructures. The MD simulations suggest that the local orientation of individual grains with the loading axis plays a critical role in determining the ability of grains to nucleate either compression twins or tension twins. The twinning behavior is observed through nucleation of a pair of planar faults and lateral growth of the twins occurs through nucleation of steps along the planar faults. The kinetics of migration of steps that determine the rate of growth of twins are investigated at the atomic scales. The twin tip velocity computed at high strain rates compares well with the experimentally reported values in the literature.

## Introduction

The understanding of the deformation response of hexagonal close packed metals such as Mg, Ti and their alloys at the atomic scales is a critical challenge towards the realization of next-generation lightweight metallic materials for applications in aerospace, automotive and defense industries. The challenge is attributed to the understanding of the role of microstructure and deformation twinning in microstructures comprising of grain boundaries, interfaces and texture. As a result, several experimental studies have been carried out to investigate the twinning mechanisms in HCP metals. These studies suggest two commonly observed twinning modes in Mg, Ti and Co microstructures using transmission electron microscopy^1–6^ comprising of tension twins (TTs) on {10$\bar{1}$2} and {$11\bar{2}1$} planes and compression twins on {$10\bar{1}1$} and {$11\bar{2}2$} planes. The studies also report the presence of several steps/disconnections along the {$10\bar{1}1$} compression twin (CT) boundaries in Co, AZ31 Mg alloy and Mg-Gd solid solution microstructures^4,5^. The steps are reported with varying heights that can be characterized in terms of interplanar spacing and can be either sessile or glissile. The growth of twins is reported in terms of the width of the twinned region (spacing between planar faults) and observed to occur through the motion of the glissile steps along the boundary^7^. One of the striking features of the {$10\bar{1}2$} TTs and {$10\bar{1}1$} CTs is the presence of wide single layer basal stacking faults (SFs) inside the twin lamella^7,8^. The width of such non equilibrium basal SFs have been reported to be 2–3 orders of magnitude larger than the equilibrium width of SF formed by partial dislocations gliding on the basal plane. The intersection of the SFs with the CT boundary plane results in the formation of additional steps along the twin boundary which are observed to be sessile in nature. The role of deformation twinning in the dynamic deformation response of single crystal Mg along the <a> axis has also been investigated at high strain rates using the plate impact experiments^9^, wherein the shock recovered samples suggest the presence of horizontal as well as angled {$10\bar{1}2$} TTs. The average propagation velocities of the tip of the {$10\bar{1}2$} TTs in this study is reported to be in the range of ~430 m/s to ~1000 m/s for strain rates ranging from 10^3^ to 10^5^ s^−1^. While these studies provide significant insights in the presence of deformation twins and their structures, a clear understanding of the nucleation and growth mechanisms of twins at high strain rates is still missing and cannot be achieved using experiments alone.

Classical molecular dynamics (MD) simulations have the capability to investigate these mechanisms at the atomic scales at high strain rates^10–19^. For the case of HCP systems, MD simulations carried out to investigate deformation twinning in single crystal Mg nanopillars have revealed that the deformation mode changes from tension twinning (θ ≤ 45°) to compression twinning (θ > 45°) as the c-axis of the single crystal nanopillar is rotated away from the loading axis^20^. Similarly, MD simulations of uniaxial tensile deformation of single crystal Mg with an initial nanovoid and a nanocrack have reported the presence of {$10\bar{1}2$} TTs and {$11\bar{2}1$} TTs^21^. The analysis of the microstructure and the extraction of atomic level information from MD simulations relies on the capability of the characterization algorithm to distinguish between different structural patterns i.e. crystal structures (FCC, BCC, HCP) from disordered/amorphous regions as well as from regions of the microstructure containing different types of crystal defects (dislocations, stacking faults, twin faults, voids etc.). While there are a number of algorithms such as “common neighbor analysis” (CNA)^22^, “centrosymmetry parameter (CSP)”^23^, “coordination number” (CN)^24^, “bond angle analysis” (BAA)^25^, and “dislocation extraction algorithm” (DXA)^26,27^ which can be efficiently used to analyze FCC and BCC microstructures and extract information related to crystal defects, none of these algorithms can be applied to directly distinguish different types of twin faults in HCP microstructures. For example, the TTs and CTs in the Mg nanopillars are identified using a combination of CNA and relative orientations of the matrix and twinned regions^20^ or using a “twin orientation analysis” (TOA) method based on the orientation of the c-axis with respect to the reference undeformed configuration^21^. Similarly, the “basal plane vector” (BPV)^28^, “polyhedral template matching” (PTM)^29^ method provides information on the local orientation of the c-axis of the HCP crystal structure and enables the identification of twins in the metal. These methods render misorientation angles of the basal planes within the twinned region and enable identification of the volume fraction of the material undergoing reorientation (twinning). In addition to being computationally expensive for large data sets, these tools have to be combined with CNA to analyze the structure of the twin boundary as well as other types of defect structures (stacking faults). The lack of well-developed atomistic characterization tools for HCP microstructures limits the capabilities of MD simulations to investigate deformation twinning in HCP microstructures at the atomic scale. As a result, there are no studies that discuss the nucleation and growth mechanisms of CTs and TTs in polycrystalline Mg microstructures at high strain rates at the atomic scale. The variations in grain size, orientation of each grain with the loading direction, strain rates of deformation and the presence of grain boundaries are likely to affect the observed modes of plastic deformation and twinning and a fundamental understanding of these links is missing.

One of the aims of this paper, therefore, is to introduce the extended common neighbor analysis (E-CNA) method and demonstrate its capability to investigate the nucleation and growth mechanisms and kinetics of various types of twin faults in Mg microstructures. The MD simulations discussed here use a polycrystalline Mg microstructure as a model system to investigate the role of grain orientation with the loading direction on the observed deformation twinning behavior at high strain rates. The E-CNA method combines the coordination number and CNA for each atom to retain the simplicity, robustness and efficiency of the original CNA method (which has traditionally been limited to identification and characterization of different types of bulk phases in atomistic microstructure) and extends its capabilities by incorporating descriptors for commonly observed crystal defects in HCP materials. A detailed description of the descriptors for the commonly observed crystal defects in HCP metals as well as the methodology of E-CNA is provided below. The E-CNA method enables the investigation of the role of microstructure on the observed modes of deformation twinning in HCP microstructures at the atomic scales. This paper, therefore, demonstrates the links between the orientations of the grains with the loading axis and the mode of deformation twinning in polycrystalline Mg microstructures using MD simulations. The focus of this manuscript is therefore the demonstration of the capability of the E-CNA method to characterize the atomic scale micromechanisms and kinetics of growth of tension and compression twins in polycrystalline Mg microstructures.

## Extended Common Neighbor Analysis (E-CNA)

The extended common neighbor analysis method extends the capability of the CNA characterization method to identify various types of twin faults in HCP microstructures. The E-CNA method identifies the local crystal structure around an atom by characterizing the structural environment of each bond (with nearest neighbors) as implemented in the CNA method and combines it with the coordination number to identify commonly observed crystal defects in HCP materials (such as CTs and TTs). A set of atoms are considered nearest neighbors when they lie within a specified cutoff distance, r_c_, which is the same as used for the HCP system in CNA. Each bond of an atom is described by a set of three characteristic integers (*i*, *j*, *k*). The index *i* denotes the number of nearest neighbors that are common to the atoms forming the bond; the index *j* denotes the total number of nearest neighbor bonds formed between the common neighbors; and index *k* denotes the number of bonds in the longest chain of bonds among the common neighbors. For example, in the FCC crystal structure, each atom forms bonds with its 12 nearest neighbors and each bond has a characteristic set of *i*, *j*, *k* values (descriptor) of *421*. Similarly, the nearest neighbor bonds in the HCP structure have 6 bonds with characteristic descriptor of type *421* and 6 bonds of type *422*. However, the current capability of CNA method is only limited to differentiating between different bulk crystal structures (FCC, BCC, HCP, DC etc.) in a microstructure.

**Figure 1:**
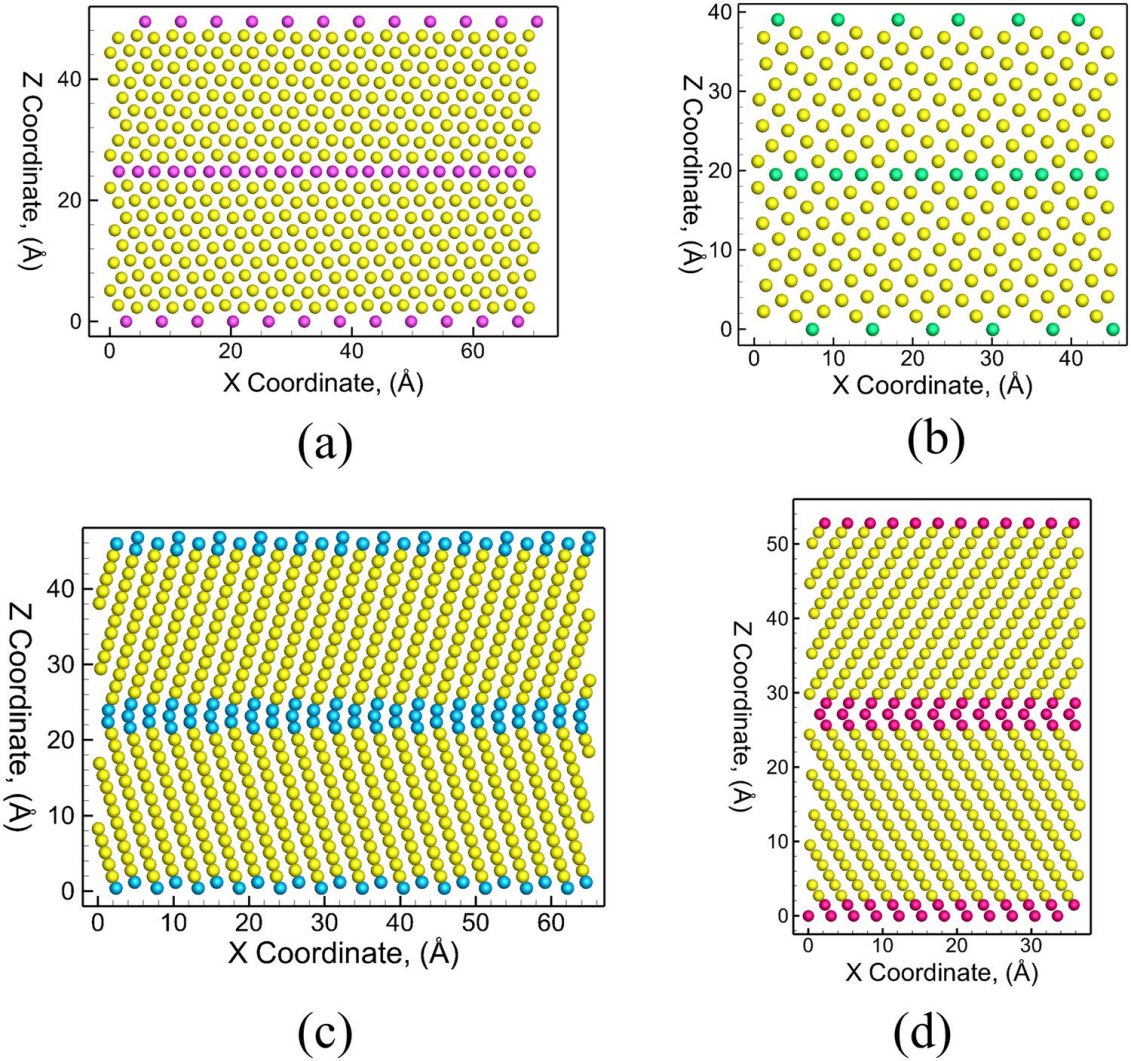
Twin boundary structure characterization of (**a**) {$10\bar{1}1$} CT, (**b**) {$10\bar{1}2$} TT, (**c**) {$11\bar{2}1$} TT, and (**d**) {$11\bar{2}2$} CT characterized using extended-common neighbor analysis. Here, atoms are colored yellow for bulk HCP stacking and dark blue for disordered atoms, purple for {$10\bar{1}1$} CT, cyan for {$10\bar{1}2$} TT, light blue for {$11\bar{2}1$} TT and pink for {$11\bar{2}2$} CT.

The E-CNA method extends this capability by identifying the descriptors for the {$10\bar{1}1$} and {$11\bar{2}2$} CTs, and the {$10\bar{1}2$}, {$11\bar{2}1$} TTs in HCP metals. The CNA analysis suggests descriptors of types *301, 312, 421, 422, 432, 412, 542*, and *551* for nearest neighbor bonds for atoms that form the CTs and TTs. These descriptors enable a direct identification of the four twins (2 CTs and 2 TTs) in HCP metals using the E-CNA method as shown in Fig. 1(a–d). Here, atoms are colored yellow for bulk HCP stacking and dark blue for disordered atoms, purple for {$10\bar{1}1$} CT, cyan for {$10\bar{1}2$} TT, light blue for {$11\bar{2}1$} TT and pink for {$11\bar{2}2$} CT. A comparison of characterization of the as-created {$10\bar{1}1$}, {$10\bar{1}2$}, {$11\bar{2}1$} and {$11\bar{2}2$} twin boundary structures using CN, CSP, CNA and E-CNA method are shown in Figs S1–S4 of the supplemental information. It can be seen from Figs S1–S4 that while none of the existing characterization algorithms enable the distinct characterization of these defect structures, the E-CNA method is able to clearly distinguish the four twin boundary structures. A comprehensive list of the descriptors for the various types of bonds for the CTs and TTs is provided in Table 1 along with the coordination numbers of the atoms that form the CTs and TTs. For example, the {$10\bar{1}1$} CT consists of atoms with two different coordination numbers (i.e. CN = 11 and CN = 13). The 11 coordinated atoms have 3 nearest neighbor bonds of type *421*, 6 nearest neighbor bonds of type *422*, and 2 nearest neighbor bonds of type *301*, whereas the 13 coordinated atoms have 5 nearest neighbor bonds of type *421*, 6 nearest neighbor bonds of type *422*, and 2 nearest neighbor bonds of type *542*.

**Table 1:** A list of descriptors (*ijk*) used in extended common neighbor analysis (E-CNA) method for ideal FCC, HCP crystal structures as well as {$10\bar{1}1$}, {$11\bar{2}2$} compression twins (CTs) and {$10\bar{1}2$}, {$11\bar{2}1$} tension twins (TTs).

E-CNA *ijk*	FCC	HCP	{$\mathrm{10}\bar{{\bf{1}}}1$} CT		{$\mathrm{11}\bar{{\bf{2}}}2$} CT		{$\mathrm{10}\bar{{\bf{1}}}2$} TT		{$\mathrm{11}\bar{{\bf{2}}}1$} TT		
	(12)	(12)	(11)	(13)	(11)	(12)	(11)	(13)	(12)	(12)	(12)
301	0	0	**2**	0	**1**	**2**	0	0	0	0	**1**
312	0	0	0	0	**3**	0	0	0	**1**	0	**1**
421	**12**	**6**	**3**	**5**	**2**	**2**	**5**	**7**	**3**	**2**	**1**
422	0	**6**	**6**	**6**	**4**	**4**	**4**	**4**	**3**	**6**	**6**
432	0	0	0	0	**1**	**4**	**2**	0	**3**	**2**	**1**
412	0	0	0	0	0	0	0	**2**	**1**	**2**	0
542	0	0	0	**2**	0	0	0	0	**1**	0	**1**
551	0	0	0	0	0	0	0	0	0	0	**1**

The E-CNA uses the coordination number (CN) for each structure in addition to the descriptors to identify TTs and CTs in the microstructure.

The same set of descriptors hold true for twin faults in other HCP metals (Ti, Zr etc.). In addition, the descriptors for these defect structures are observed to be independent of the choice of interatomic potential. For example, the as-created twin boundary structures of {10–11}, {11–22} CTs as well as {10–12}, {11–21} TTs relaxed using the MEAM potential^30^ are also accurately characterized using the same set of descriptors as shown in Figs S1(e)–S4(e). The E-CNA method is as computationally efficient, robust and simple as the CNA method and can be used to efficiently analyze large datasets generated in MD simulations (containing billions of atoms). In addition, the E-CNA method can also be generalized to other crystal structure types as well as other defect structures by creating a database of such structures and extracting descriptors for bond environments from those structures. The links between the orientations of the grains with the loading axis and the mode of deformation twinning in polycrystalline Mg microstructures using MD simulations as well as the atomic scale mechanisms of growth of TTs and CTs is discussed below.

## Results and Discussions

**Figure 2:**
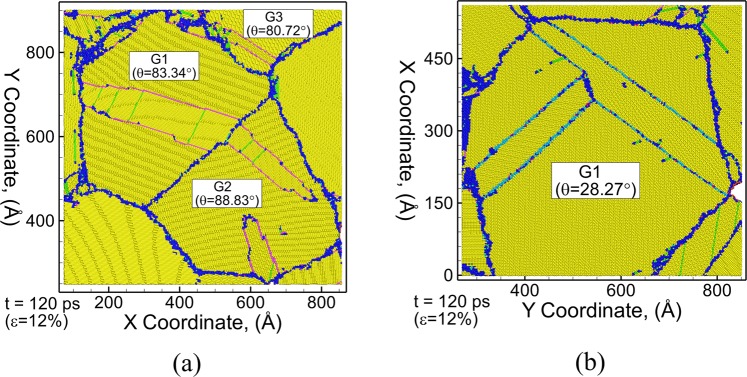
Microstructural snapshots of thin section (3 nm) of polycrystalline Mg perpendicular to Z-direction at a time (strain) of 120 ps (12%) during uniaxial tensile stress loading along (**a**) Z-direction and (**b**) X-direction. The atoms are colored using E-CNA method. Bulk HCP atoms are colored yellow, grain boundary/disordered atoms are colored dark blue, FCC atoms (basal stacking fault) are colored green, purple atoms represent {${\rm{10}}\bar{1}1$} CT boundary, light blue atoms represent {${\rm{11}}\bar{2}1$} TT boundary and red atoms represent surface/voids.

Deformation twinning in Mg microstructures is investigated by deforming a polycrystalline Mg system comprising of an average grain size of 50 nm under uniaxial tensile stress loading at a strain rate of 10^9^ s^−1^. The simulations are carried out for loading in the X, Y and Z directions to enable all active twinning modes for each grain in the microstructure. Figure 2(a,b) shows two example snapshots of a thin (3 nm thick) section of the polycrystalline microstructure for loading along the Z-direction and X-direction respectively. The microstructure in Fig. 2(a) shows three grains (grain 1 (G1), grain 2 (G2) and grain 3 (G3)) that deform by nucleation of {$10\bar{1}1$} CTs, wherein the c-axis of these grains have an initial local orientation of 83.34°, 88.83° and 80.72° respectively with the loading (Z-axis) axis. Similarly, the microstructure in Fig. 2(b) depicts the nucleation of {$11\bar{2}1$} TTs in grain G1 when the c-axis is oriented at an angle of 28.27° with the loading axis (X-axis). The role of loading orientation with the c-axis of the grains on the nucleation of CTs and TTs agrees well with previously reported MD simulations for single crystal Mg nanopillars that suggest the nucleation of {$11\bar{2}1$} TTs as the primary deformation mode when the c-axis is oriented at an angle of 15° < θ < 45° with the loading axis, while {$10\bar{1}1$} CTs are favored when c-axis is oriented at an angle of 60° < θ < 90° with the loading axis^20^. The E-CNA method can now be used to characterize the dynamic evolution of twins and identify the growth mechanisms as discussed below.

**Figure 3:**
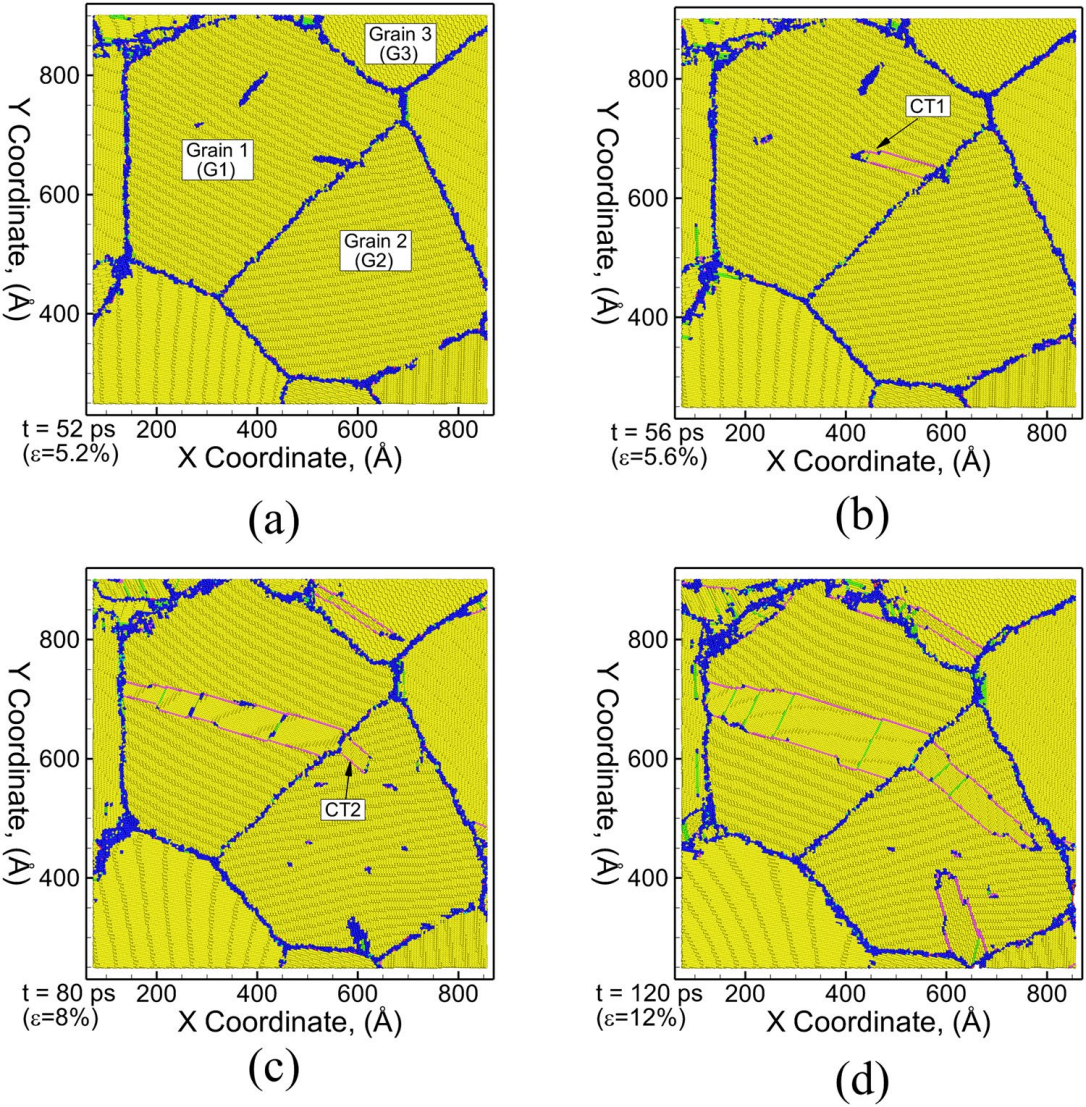
Microstructural evolution of thin section (3 nm) of polycrystalline Mg during uniaxial tensile stress loading at strain rate of 10^9^ s^−1^ at a time (strain) of (**a**) 52 ps (5.2%), (**b**) 56 ps (5.6%), (**c**) 80 ps (8%) and (**d**) 120 ps (12%). The atoms are colored using E-CNA method. Bulk HCP atoms are colored yellow, grain boundary/disordered atoms are colored dark blue, FCC atoms (basal stacking fault) are colored green, purple atoms represent {${\rm{10}}\bar{1}1$} CT boundary, light blue atoms represent {${\rm{11}}\bar{2}1$} TT boundary and red atoms represent surface/voids.

The growth mechanisms of CTs shown in Fig. 2(a) are analyzed using a series of snapshots at different times (strains) during uniaxial tensile loading in Fig. 3. The {$10\bar{1}1$} CT is observed to nucleate at the grain boundary between grains G1 and G2 at a time of 52 ps (*ε* = 5.2% strain) as shown in Fig. 3(a). Once nucleated, the {$10\bar{1}1$} CT grows as a pair of planar faults in the direction towards the other end of the grain and also grows in the lateral direction as shown in Fig. 3(b). The tip of the twin boundary CT1 propagates until it meets the grain boundary at the other end of the grain. The grain boundary acts as a barrier for further twin growth in the direction of propagation of the tip as shown in Fig. 3(c). Further straining, however, leads to the nucleation of compression twin CT2 in grain G2 as shown in Fig. 3(c) and is attributed to the favorable grain orientation of G2 with the loading axis (θ = 88.83°). Further straining limits the growth of CT1 only in the lateral direction leading to an increase in the width of the twin fault (distance between the two planar fronts of the twin boundary) as can be seen in Fig. 3(d) at a time of 120 ps (12% strain) for CT1. The twin CT2 continues to grow towards the opposite end of the grain.

**Figure 4:**
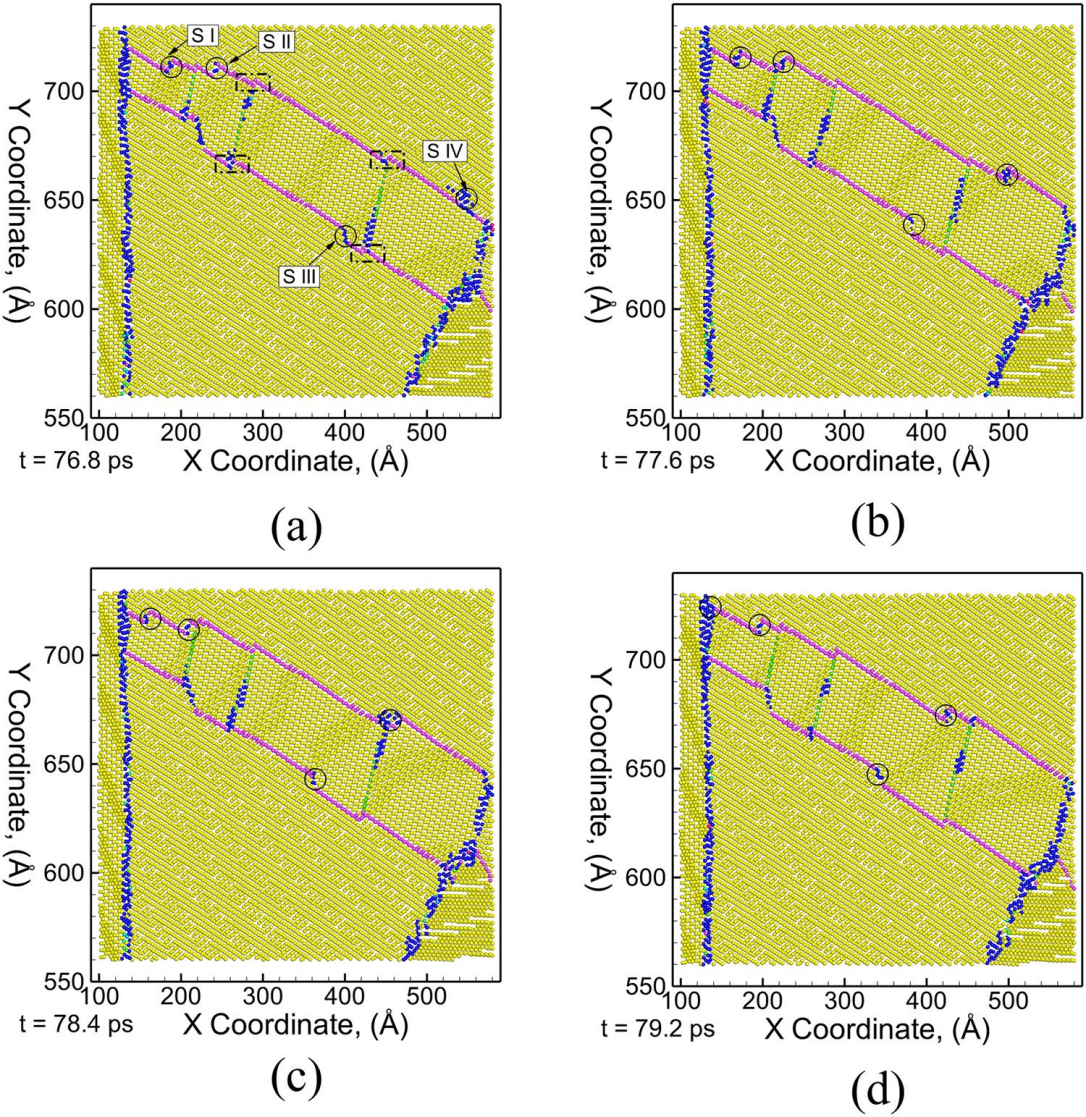
Microstructural snapshots depicting the growth of {$10\bar{1}1$} CT by movement of steps along the twin boundary during uniaxial tensile stress loading at a strain rate of 10^9^ s^−1^ at a time (strain) of (**a**) 76.8 ps (7.68%), (**b**) 77.6 ps (7.76%), (**c**) 78.4 ps (7.84%) and (**d**) 79.2 ps (7.92%). The atoms are colored using E-CNA method. Bulk HCP atoms are colored yellow, grain boundary/disordered atoms are colored dark blue, FCC atoms (basal stacking fault) are colored green, purple atoms represent {${\rm{10}}\bar{1}1$} CT boundary, light blue atoms represent {${\rm{11}}\bar{2}1$} TT boundary and red atoms represent surface/voids.

A detailed analysis of the structure of the CT1 twin boundary and mechanisms of lateral twin growth in grain G1 is shown using a series of snapshots in Fig. 4. One of the key features of CTs nucleated in different grains of polycrystalline microstructures is the presence of wide single layer basal stacking faults extending from one planar front to the other planar front of the TB as well as the presence of sessile (marked by dashed rectangle in Fig. 4(a)) and glissile steps (SI, SII, SIII and SIV) along the twin boundary. Similar evidences of {$10\bar{1}1$} CTs with stepped interface and high density of basal and {$10\bar{1}1$} pyramidal stacking faults inside the twin lamella have also been reported in experimental TEM studies of AZ31 Mg alloy, pure Co and pure Ti deformed under quasi-static loading^4,5,7^. The sessile steps are observed to be associated with the presence of basal stacking faults and are pinned at the point of intersection of the stacking fault with the twin boundary. A detailed movie showing the nucleation and growth mechanism of the {$10\bar{1}1$} twin fault (CT1) in grain G1 is provided in the Supplementary Material (Movie S1). The lateral growth of the twins occurs through the nucleation of steps that propagate towards the opposite grain boundary. The nucleation of glissile steps (SIV) is observed to occur at the grain boundary. Once nucleated, the propagation of these steps along the twin boundary results in the thickening of the entire twinned region. Since the twin boundary structure comprises of an atomically thin layer (as shown in Fig. 1(a)), the growth in the width (thickness) of the twinned region in the lateral region is observed to occur through nucleation of steps at the grain boundary. The presence and structure of the grain boundary is likely to assist in the nucleation of these steps. However, a more detailed analysis is needed to understand the role of grain boundaries in the nucleation phenomena. As a result, at any given time, the width between the planar faults is the largest at the base of the twin (origin) and the smallest at the end of the fault (opposite grain boundary). For example, the lateral growth of the twin causes nucleation of step SIV near the grain boundary as shown in Fig. 4(a). Once nucleated, the glissile steps migrate towards the opposite grain boundary resulting in an increase in width of the twinned region as well as the width of the basal stacking fault connecting the two planar fronts of the twin boundary.

**Figure 5:**
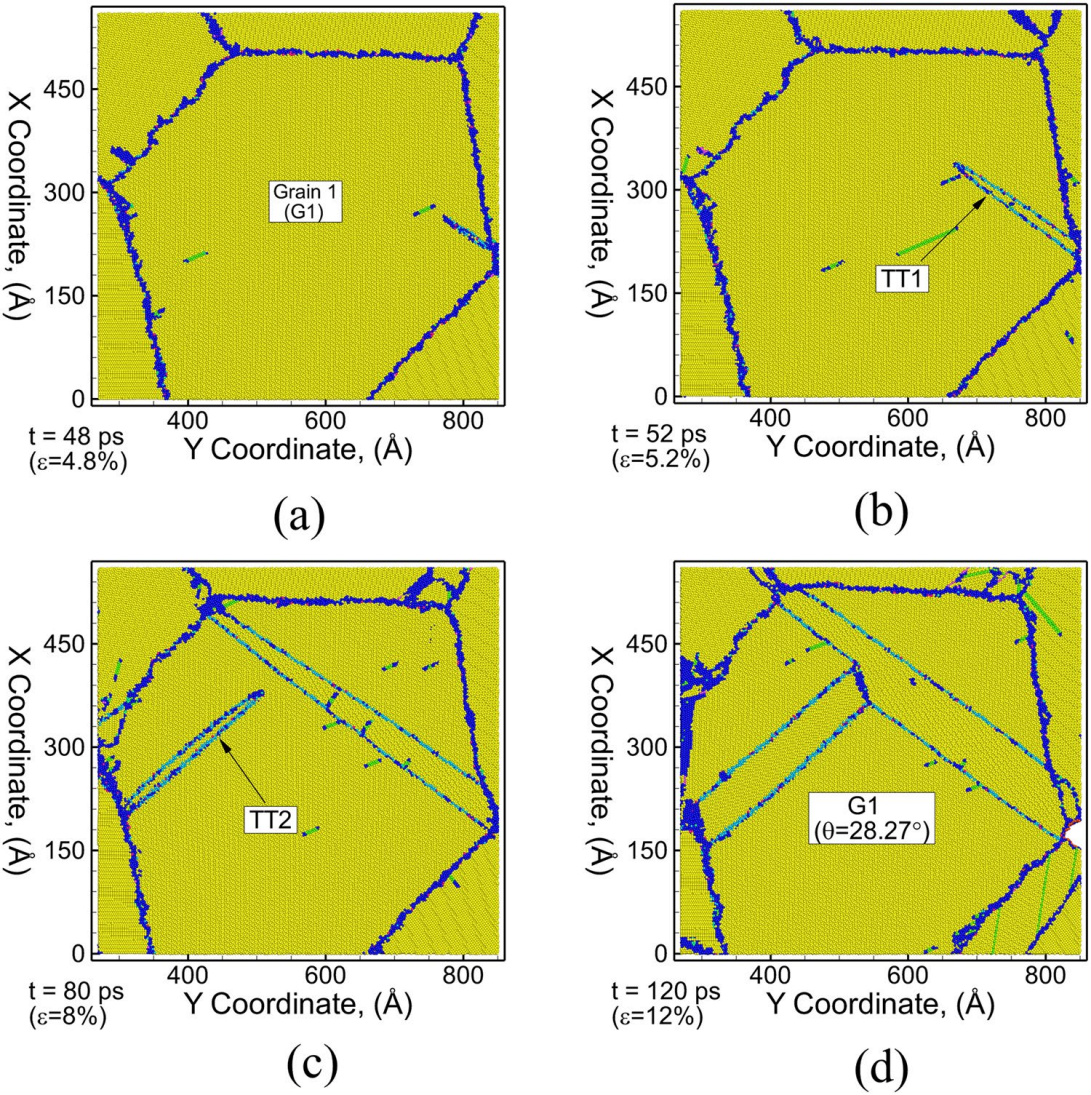
Microstructural evolution of thin section (3 nm) of polycrystalline Mg during uniaxial tensile stress loading at strain rate of 10^9^ s^−1^ at a time (strain) of (**a**) 48 ps (4.8%), (**b**) 52 ps (5.2%), (**c**) 80 ps (8%) and (**d**) 120 ps (12%). The atoms are colored using E-CNA method. Bulk HCP atoms are colored yellow, grain boundary/disordered atoms are colored dark blue, FCC atoms (basal stacking fault) are colored green, purple atoms represent {${\rm{10}}\bar{1}1$} CT boundary, light blue atoms represent {${\rm{11}}\bar{2}1$} TT boundary and red atoms represent surface/voids.

Similarly, the growth mechanism of {$11\bar{2}1$} TTs is investigated using a series of snapshots of a thin (3 nm) section of the polycrystalline microstructure perpendicular to the Z-direction for uniaxial tensile loading along X-direction as shown in Fig. 5. The {11–21} TT (TT1) also nucleates as a pair of planar faults from one end of the grain boundary in grain G1 and propagates towards the opposite grain boundary as shown in Fig. 5(a,b). As the twin propagates towards the grain boundary, it grows both in the direction of motion of the twin tip as well as in lateral direction. Unlike {$10\bar{1}1$} CT, the {$11\bar{2}1$} TT boundaries do not grow through nucleation of steps and also do not contain single layer basal stacking faults inside the twinned region. Once the twin reaches the other end of grain G1, the grain boundary act as a barrier for further twin growth in the direction of motion of the twin tip due to change in orientation of the grains across the grain boundary. Thus, TT1 can only grow in the lateral direction which is evident by an increase in width of the TT1 as shown in Fig. 5(c,d). A second TT (TT2) is also observed to nucleate in the same grain at a later stage during tensile deformation and propagates towards TT1 as shown in Fig. 5(c). The continued deformation results in the intersection of twins TT2 and TT1 that restricts the growth of the TT2 twin in the direction of motion of the twin tip and only allows it to grow in the lateral direction as shown by the increase in width of the twinned region in Fig. 5(d).

**Figure 6:**
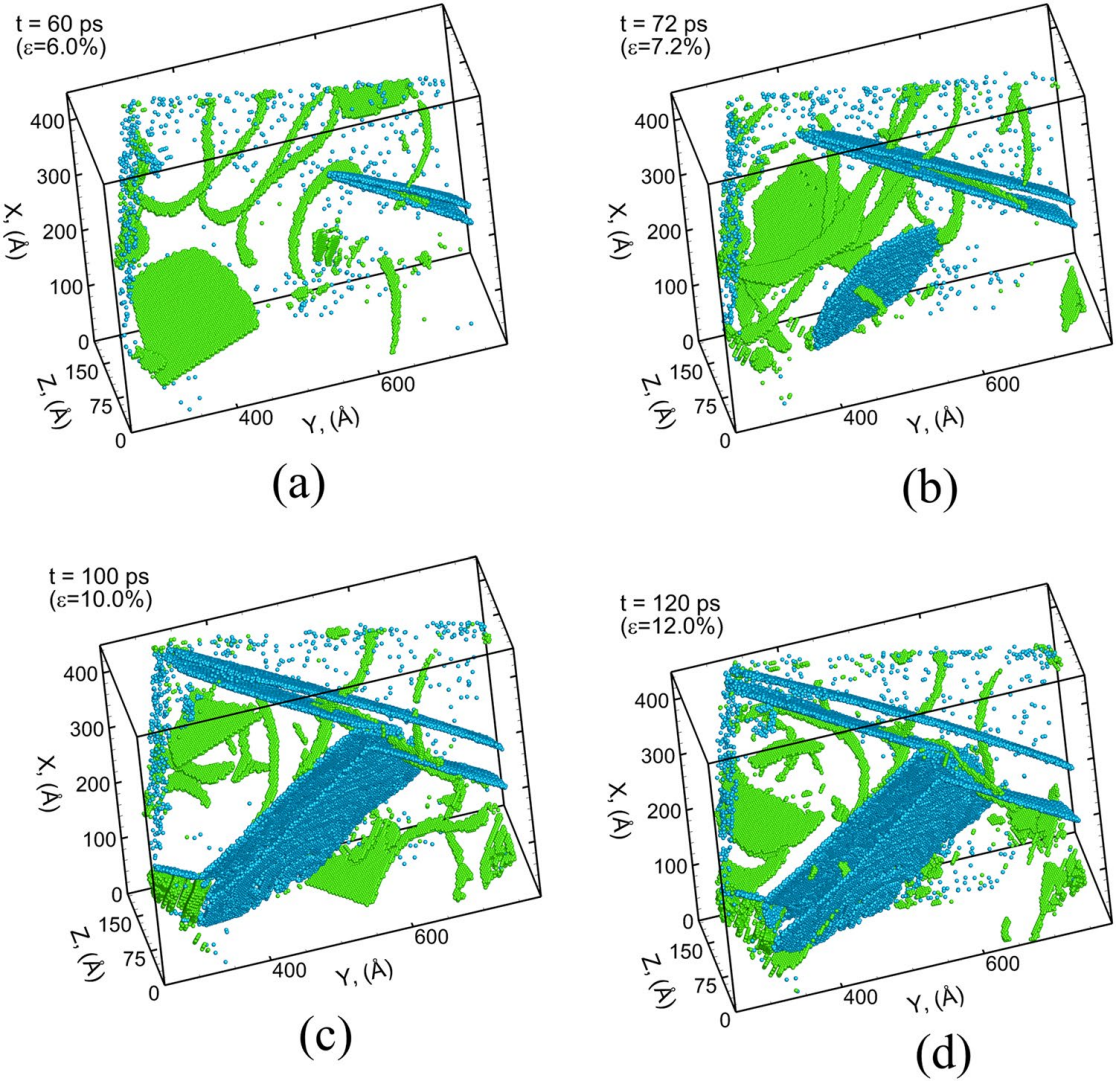
Microstructural evolution of Grain 1 (G1) of polycrystalline Mg during uniaxial tensile stress loading at strain rate of 10^9^ s^−1^ at a time (strain) of (**a**) 60 ps (6%), (**b**) 72 ps (7.2%), (**c**) 100 ps (10%) and (**d**) 120 ps (12%). The atoms are colored using E-CNA method. Only, atoms belonging to $\{{\rm{11}}\bar{2}1\}$ TT (light blue) and basal stacking faults (green) are shown. Other types of atoms are blanked out to clearly visualize the interaction between basal stacking faults and TT1.

A detailed analysis of the interaction of tension twin TT1 with the basal stacking faults is shown using a series of snapshots in Fig. 6. The bulk HCP atoms as well as the disordered/grain boundary atoms are not shown in the snapshots in order to clearly visualize the interaction of the twin boundary with basal stacking faults. The basal stacking faults are observed to nucleate in the grain prior to the nucleation of the tension twin TT1. The nucleated twin TT1 grows towards the other end of grain resulting in the intersection of the basal stacking faults with the twin boundary as shown in Fig. 6(a). These intersections result in the transmission of the basal stacking faults from the matrix to the twinned region bounded by the two planar twin boundaries as shown in Fig. 6(b). These stacking faults are observed to be different from the single layer (I1 type) stacking faults present within {${\rm{10}}\bar{1}1$} CT as shown earlier. These are two layer stacking faults (I2) bounded by two Shockley partial type dislocations (1/3 <$1\bar{1}{\rm{00}}$>) which are formed by the dissociation of a Perfect dislocation (1/3 <${\rm{11}}\bar{2}0$>) on the basal plane of Mg. Unlike {${\rm{10}}\bar{1}1$} CT, the boundaries of {${\rm{11}}\bar{2}1$} TT consist of multiple planes of atoms (3 planes as shown in Fig. 1(c)) and the growth in the width of {${\rm{11}}\bar{2}1$} TT doesn’t occur by the nucleation of steps along the twin boundary. The growth in the thickness is observed to happen through shuffling of atoms layer by layer at the boundary. The lateral growth of TT1 is accompanied by in increase in the width of the basal stacking fault bounded by the two boundaries of the twin fault as shown in Fig. 6(c,d). Similar interaction and the transmission of the basal dislocation from the matrix into the $\{{\rm{11}}\bar{2}1\}$ TT boundary have been reported in previous MD simulations of single crystal Mg nanopillar^20^. A detailed movie showing the showing the propagation, growth and interaction mechanism of the {$11\bar{2}1$} tension twin fault with basal stacking faults is provided in the Supplementary Material (Movie S2).

**Figure 7:**
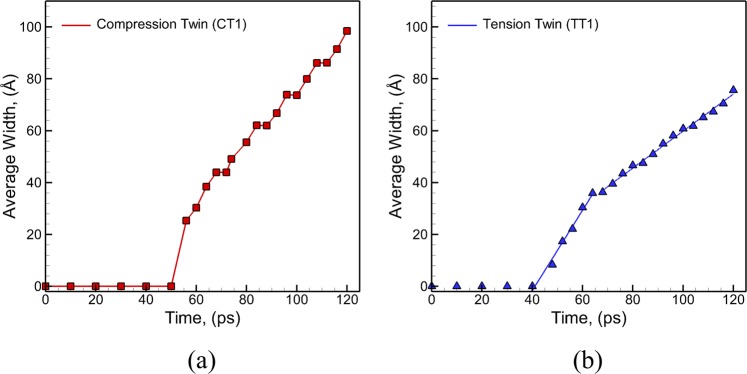
Variation of average width of the twin fault (**a**) {${\rm{10}}\bar{1}1$} CT1 and (**b**) {${\rm{11}}\bar{2}1$} TT1 as a function of time during uniaxial tensile stress loading at a strain rate of 10^9^ s^−1^.

Thus, the E-CNA method allows to investigate the kinetics of growth of CTs and TTs in polycrystalline Mg microstructures. The series of snapshots discussed earlier in Fig. 3 can also be used to track the position of twin tip as a function of time and calculate the average velocity of the tip of the twin boundary. The twin tip velocity for CT boundary marked as CT1 is calculated to be 1549 ± 327 m/s at a strain rate of 10^9^ s^−1^. The series of snapshots in Fig. 4 can be used to track the position of the glissile steps along the twin boundary resulting in an average velocity of 1915 ± 396 m/s at a loading strain rate of 10^9^ s^−1^. The plot showing the variation of average distance between the two planar fronts (average width) of compression twin CT1 at the base (origin) of the twin fault as a function of time is shown in Fig. 7(a). It can be seen that the increase in the width of the twin fault occurs in a step-wise manner which indicates that the lateral growth of compression twin CT1 is guided by the nucleation and propagation of the steps along the twin boundary plane. Similarly, the average velocity of the tip of tension twin TT1 is calculated using a series of snapshots in Fig. 5 to be 2371 ± 364 m/s for uniaxial tensile loading at strain rate of 10^9^ s^−1^. The tip velocities of the horizontal and angled {$10\bar{1}2$} TTs in plate impact experiments of polycrystalline AZ31 Mg alloy are calculated to be ~1000 m/s and ~430 m/s at a strain rate of ~10^3^–10^5^ s^−1^ ^9^. These values are lower than the MD predicted values computed at ~4 orders of magnitude higher rates of loading. However, this study has not been able to find experimental values in the literature for the propagation velocities of {$11\bar{2}1$} TTs. In addition, the average distance between the two planar fronts of TT1 near the originating grain boundary is tracked as a function of time and plotted in Fig. 7(b). Unlike $\{10\bar{1}1\}\,\,\,$CT1, the lateral growth (increase in the width) of TT1 occurs in a continuous manner in two distinct stages. It can be seen that the average rate of growth of the width of the twin boundary TT1 decreases after the tip of the tension twin TT1 reaches the other end of the grain (at a time of ~68 ps). The mode of lateral growth (shuffling of atoms layer by layer) observed for the TT does not allow large displacements and hence renders slower growth of the TT in the lateral direction as compared to the CT that involves nucleation of steps.

Thus, the E-CNA method allows an atomic level characterization of the mechanisms of growth of TTs and CTs as well as investigation of growth kinetics of these twins in polycrystalline Mg microstructures. The current focus is to investigate the role of microstructure and loading conditions on the ability of grains to undergo deformation twinning. The effect of grain size and strain rates on the atomic scale nucleation mechanisms of TTs and CTs in polycrystalline microstructures will be the focus of a future manuscript.

## Conclusions

The capabilities of “common neighbor analysis” method is extended to identify the atomic scale environments that represent compression twins and tension twins in HCP microstructures. The capability of this “extended common neighbor analysis” method to characterize the evolution of deformation twins is demonstrated using polycrystalline Mg microstructures subjected to uniaxial tensile stress deformation using MD simulations. The deformation response of polycrystalline Mg microstructures is observed to be dominated by the nucleation and growth of compression and tension twins wherein the orientation of the c-axis of the grain with the loading axis is observed to play a critical role in determining the type of twin variant (compression twin vs tension twin) nucleated in each grain. The CTs and TTs are observed to nucleate as a pair of planar faults at one end of the grain and grow towards the other end of the grain. This migration of the twin tip is attributed to the lateral growth of the twinned region that occurs through nucleation of steps along the planar faults. These insights allow for the development of better predictive models at the continuum scale to achieve an improved understanding of mechanisms of nucleation, evolution and interaction of defects in HCP microstructures. The E-CNA method allows for developing atomic level understanding of the twin boundary structure for different types of compression and tension twins. It can be combined with other local orientation analysis methods, such as, BPV, TOA and PTM, to investigate the evolution of volume fraction of different types of deformation twins in the microstructure. It should be noted that the deformation behavior of the polycrystalline Mg predicted by MD simulations depends on the choice of the interatomic potential. However, irrespective of the choice of the interatomic potential, the E-CNA method opens up opportunities to systematically investigate various aspects of the nucleation and growth mechanisms of twin faults such as volume fraction of material undergoing twinning, rate of growth of the twin boundary, twin tip velocity, interface structure of the twin boundary etc. in polycrystalline HCP microstructures. Currently, the E-CNA method is being implemented in LAMMPS and will be added as a compute in the upcoming versions of LAMMPS.

## Methods

### Polycrystalline microstructures

The initial polycrystalline Mg microstructure is created with dimensions of 100 nm × 100 nm × 100 nm and an average grain size of 50 nm (~43 million atoms) using the “Voronoi tessellation method”^31^ consisting of 15 randomly oriented grains with periodic boundary conditions along all the three directions. The as-created polycrystalline system is equilibrated at zero pressure and 300 K prior to deformation using NPT ensemble.

### Interatomic potentials

The MD simulations are carried out using a variant of the original Sun embedded atom method (EAM) potential (referred as “new Sun” potential in this paper)^32^.

### Extended Common Neighbor Analysis (E-CNA) Method

The E-CNA method extends the capabilities of original CNA^22^ to characterize defect structures in HCP microstructures by incorporating descriptors for commonly observed twin faults. The manuscript uses E-CNA method to characterize the nucleation and evolution of different types of twins (tension and compression twins) in the microstructure during uniaxial tensile deformation simulations of polycrystalline Mg. The detailed comparison of characterization of microstructures using E-CNA with other methods is provided in the supplementary material.

### Uniaxial Tensile Stress Simulation Setup

The MD simulations of uniaxial tensile stress loading are carried out using LAMMPS^33^ at a constant strain rate of 10^9^ s^−1^. Due to the small number of grains in the microstructure, several simulations are carried out to investigate deformation twinning under uniaxial tensile stress loading along the X (σ_x_ ≠ 0, σ_y_ = σ_z_ = 0), Y (σ_y_ ≠ 0, σ_x_ = σ_z_ = 0) and Z (σ_z_ ≠ 0, σ_x_ = σ_y_ = 0) directions in order to generate more statistics on the variation of the orientation of c-axis of the grains with the loading axis. The local orientation of the c-axis of each grain with the loading axis in the initial microstructure as well as in the deformed microstructure is determined using basal plane vector (BPV) analysis^28^ implemented in LAMMPS. All the MD simulations are carried out using a time-step of 2 fs and temperature is allowed to evolve during the simulation.

## Supplementary information

Supplementary Information

S1

S2

## Data Availability

Any data that is requested by the Editors and Reviewers will be made available upon request.
