# Supplementary material for: Deformation Twinning in Polycrystalline Mg Microstructures at High Strain Rates at the Atomic Scales
Source: Sci Rep. 2019 Mar 5;9:3550. doi: 10.1038/s41598-019-39958-w (PMC6400955; doi:10.1038/s41598-019-39958-w)
Supplement: Supplementary file 1 — Supplementary Information [file 41598_2019_39958_MOESM1_ESM.docx]

SUPPLEMENTAL INFORMATION, Figures and figure captions

Title: Deformation Twinning in Polycrystalline Mg Microstructures at High Strain Rates at the Atomic Scales

*Garvit Agarwal^1^ and Avinash M. Dongare^1,*^*

^1^ Department of Materials Science and Engineering and Institute of Materials Science, University of Connecticut, Storrs, Connecticut 06269, USA

*** Corresponding Author:**

Avinash M. Dongare

Materials Science and Engineering, University of Connecticut, Storrs, CT-06084

Email: [dongare@uconn.edu](mailto:dongare@uconn.edu)

S1: Extended Common Neighbor Analysis (E-CNA):

The E-CNA method extends this capability by identifying the descriptors for the {$\mathbf{10}\bar{\mathbf{1}}\mathbf{1}$} and {$\mathbf{11}\bar{\mathbf{2}}\mathbf{2}$} CTs, and the {$\mathbf{10}\bar{\mathbf{1}}\mathbf{2}$}, {$\mathbf{11}\bar{\mathbf{2}}\mathbf{1}$} TTs in HCP metals. A comparison of characterization of the as-created {$\mathbf{10}\bar{\mathbf{1}}\mathbf{1}$}, {$\mathbf{10}\bar{\mathbf{1}}\mathbf{2}$}, {$\mathbf{11}\bar{\mathbf{2}}\mathbf{1}$} and {$\mathbf{11}\bar{\mathbf{2}}\mathbf{2}$} twin boundary structures using CN, CSP, CNA and E-CNA method are shown in Figure S1-S4.


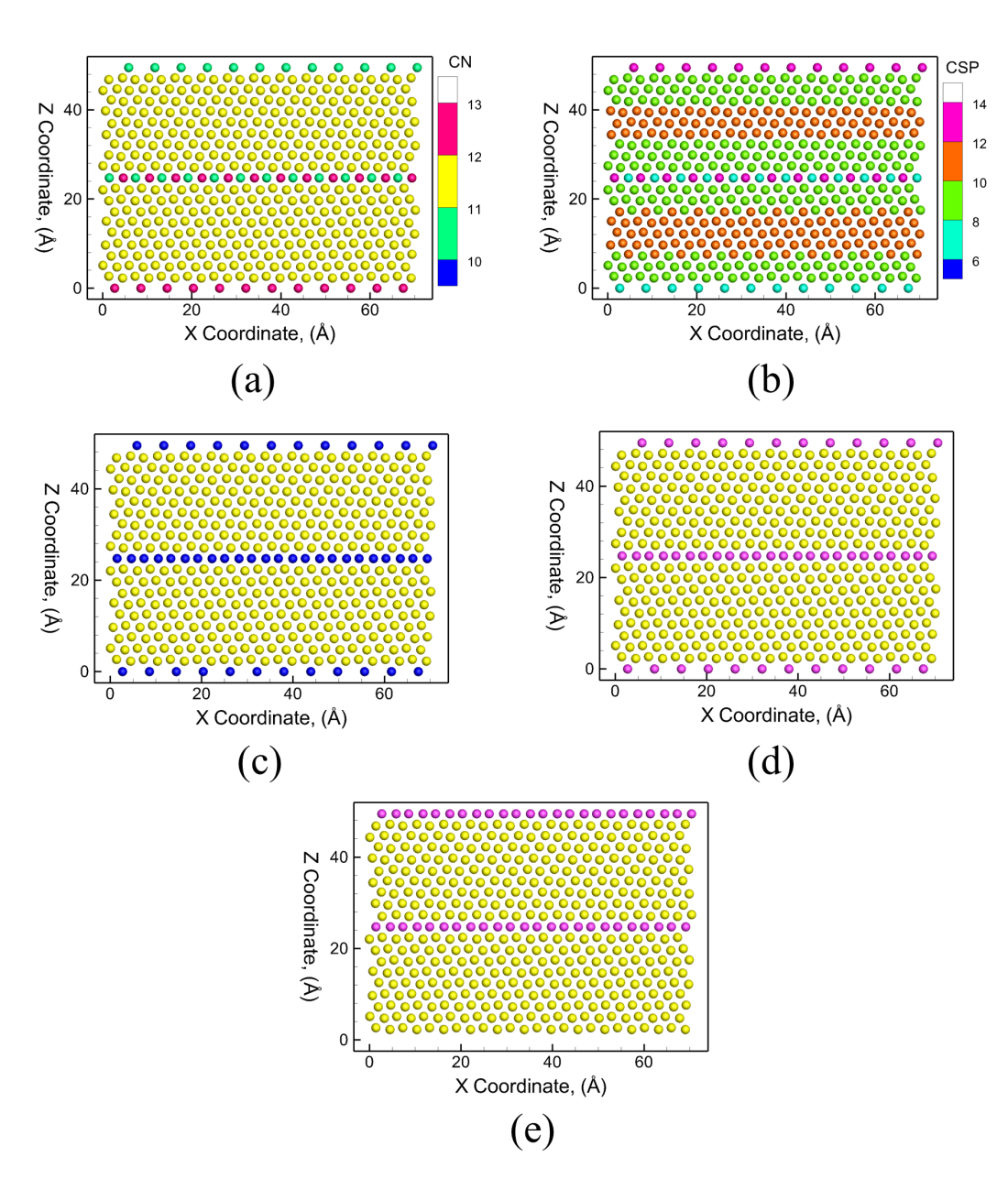


**Figure S1:** Twin boundary structure of {$10\bar{1}1$} CT as predicted by Sun potential characterized using (a) coordination analysis (CN), (b) centrosymmetry parameter (CSP), (c) common neighbor analysis (CNA), (d) extended-common neighbor analysis (E-CNA) and (e) MEAM potential predicted twin boundary structure characterized using E-CNA. The coloring legend for CN and CSP are given on the right in each image. The atoms are colored yellow for bulk HCP stacking and dark blue for disordered atoms in case of CNA. Similarly in case of E-CNA, the atoms are colored yellow for bulk HCP stacking, purple for {$10\bar{1}1$} CT, cyan for {$10\bar{1}2$} TT, light blue for {$11\bar{2}1$} TT and pink for {$11\bar{2}2$} CT


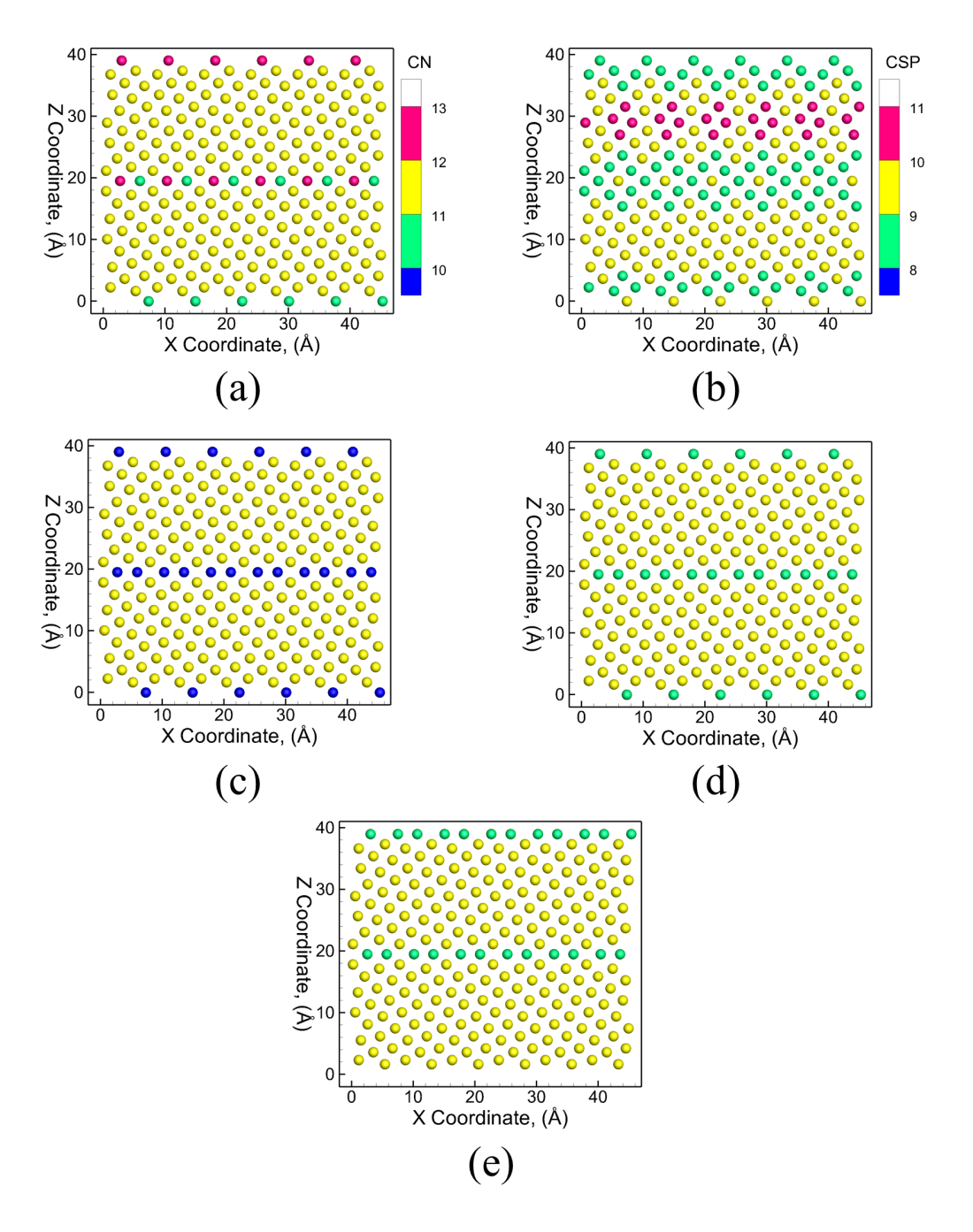


**Figure S2:** Twin boundary structure of {$10\bar{1}2$} TT as predicted by Sun potential characterized using (a) coordination analysis (CN), (b) centrosymmetry parameter (CSP), (c) common neighbor analysis (CNA), (d) extended-common neighbor analysis (E-CNA) and (e) MEAM potential predicted twin boundary structure characterized using E-CNA. The coloring legend for CN and CSP are given on the right in each image. The atoms are colored yellow for bulk HCP stacking and dark blue for disordered atoms in case of CNA. Similarly in case of E-CNA, the atoms are colored yellow for bulk HCP stacking, purple for {$10\bar{1}1$} CT, cyan for {$10\bar{1}2$} TT, light blue for {$11\bar{2}1$} TT and pink for {$11\bar{2}2$} CT

**
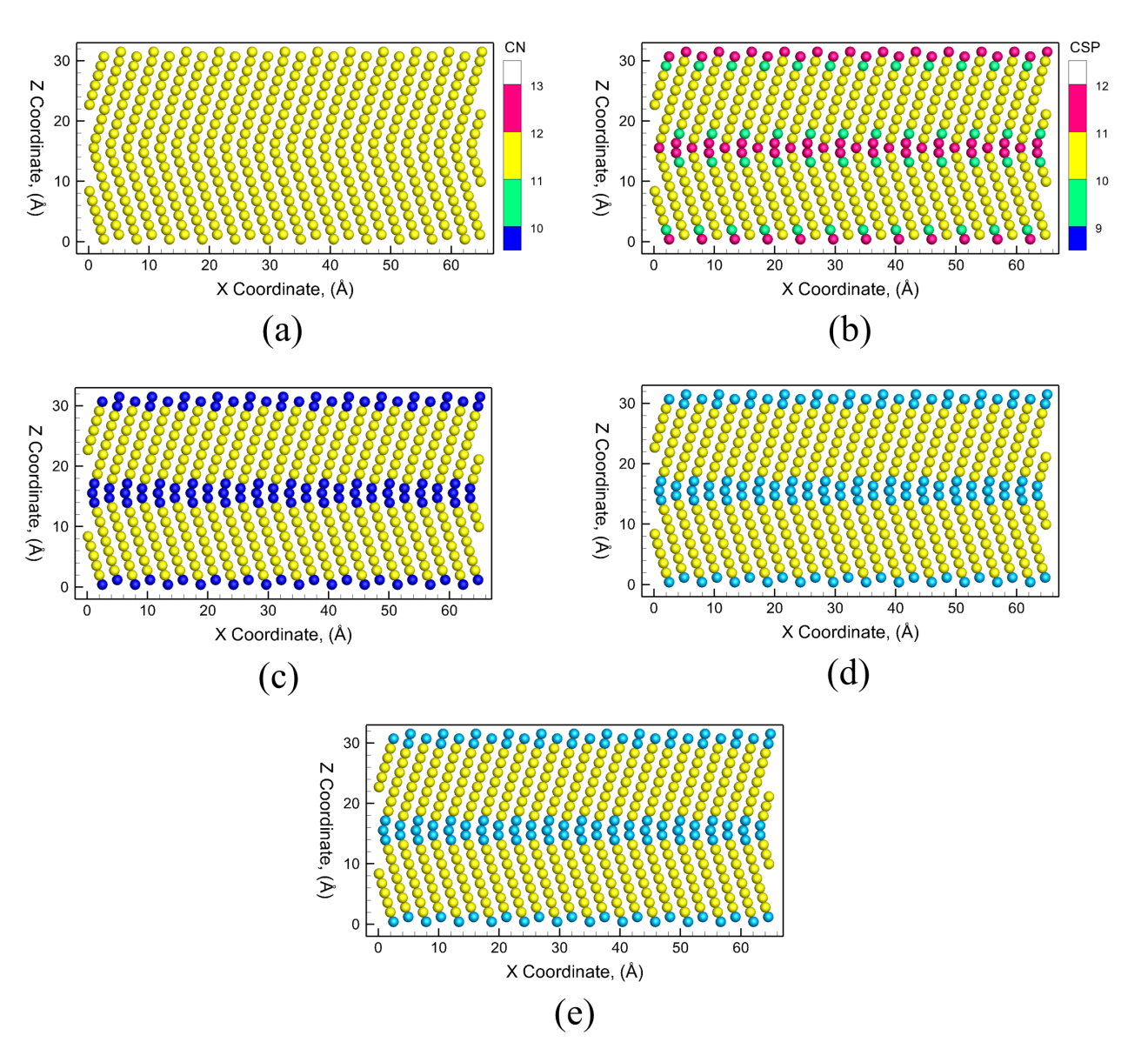
**

**Figure S3:** Twin boundary structure of {$11\bar{2}1$} TT as predicted by Sun potential characterized using (a) coordination analysis (CN), (b) centrosymmetry parameter (CSP), (c) common neighbor analysis (CNA), (d) extended-common neighbor analysis (E-CNA) and (e) MEAM potential predicted twin boundary structure characterized using E-CNA. The coloring legend for CN and CSP are given on the right in each image. The atoms are colored yellow for bulk HCP stacking and dark blue for disordered atoms in case of CNA. Similarly in case of E-CNA, the atoms are colored yellow for bulk HCP stacking, purple for {$10\bar{1}1$} CT, cyan for {$10\bar{1}2$} TT, light blue for {$11\bar{2}1$} TT and pink for {$11\bar{2}2$} CT

**
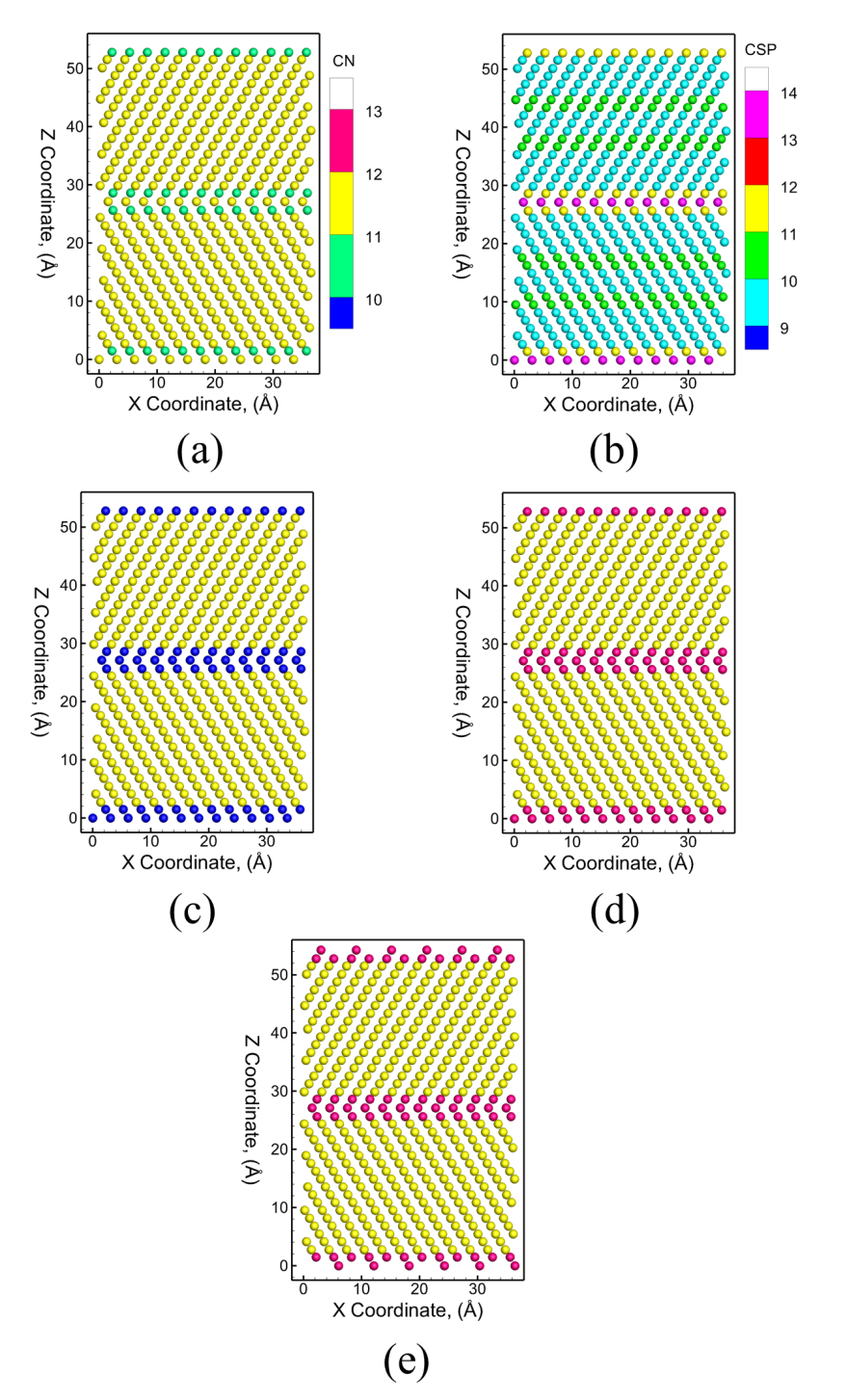
**

**Figure S4:** Twin boundary structure of {$11\bar{2}2$} CT as predicted by Sun potential characterized using (a) coordination analysis (CN), (b) centrosymmetry parameter (CSP), (c) common neighbor analysis (CNA), (d) extended-common neighbor analysis (E-CNA) and (e) MEAM potential predicted twin boundary structure characterized using E-CNA. The coloring legend for CN and CSP are given on the right in each image. The atoms are colored yellow for bulk HCP stacking and dark blue for disordered atoms in case of CNA. Similarly in case of E-CNA, the atoms are colored yellow for bulk HCP stacking, purple for {$10\bar{1}1$} CT, cyan for {$10\bar{1}2$} TT, light blue for {$11\bar{2}1$} TT and pink for {$11\bar{2}2$} CT

S2: Supplemental Movies

Movie S1: The time evolution of a thin section (3 nm) of polycrystalline Mg microstructure during uniaxial tensile stress loading at a strain rate of 10^9^ s^-1^ showing the nucleation and growth mechanism of the {$\boldsymbol{10}\bar{\boldsymbol{1}}\boldsymbol{1}$} compression twin fault. Bulk HCP atoms are colored yellow, grain boundary/disordered atoms are colored dark blue, FCC atoms (basal stacking fault) are colored green, purple atoms represent {$\text{10}\bar{\text{1}}\text{1}$} CT boundary, light blue atoms represent {$\text{11}\bar{\text{2}}\text{1}$} TT boundary and red atoms represent surface/voids

**Movie S2:** The time evolution of tension twin (TT1) in grain G1 of polycrystalline Mg microstructure during uniaxial tensile stress loading at a strain rate of 10^9^ s^-1^ showing the propagation, growth and interaction mechanism of the {$11\bar{2}1$} tension twin fault with basal stacking faults. Bulk HCP atoms and grain boundary/disordered atoms are not shown to clearly visualize interaction of twin boundary (light blue atoms) with basal stacking faults (green atoms)
